# Probiotic Activity of *Staphylococcus epidermidis* Induces Collagen Type I Production through FFaR2/p-ERK Signaling

**DOI:** 10.3390/ijms22031414

**Published:** 2021-01-31

**Authors:** Indira Putri Negari, Sunita Keshari, Chun-Ming Huang

**Affiliations:** 1 Department of Biomedical Sciences and Engineering, National Central University, Taoyuan 32001, Taiwan; indiraputri24@g.ncu.edu.tw; 2 Department of Life Sciences, National Central University, Taoyuan 32001, Taiwan; sunitakeshari827@gmail.com

**Keywords:** butyric acid, collagen type I, probiotic, *Staphylococcus epidermidis*

## Abstract

Collagen type I is a key structural component of dermis tissue and is produced by fibroblasts and the extracellular matrix. The skin aging process, which is caused by intrinsic or extrinsic factors, such as natural aging or free radical exposure, greatly reduces collagen expression, thereby leading to obstructed skin elasticity. We investigated the effective fermentation of Cetearyl isononanoate (CIN), a polyethylene glycol (PEG) analog, as a carbon source with the skin probiotic bacterium *Staphylococcus epidermidis* (*S.* *epidermidis*) or butyrate, as their fermentation metabolites could noticeably restore collagen expression through phosphorylated extracellular signal regulated kinase (p-ERK) activation in mouse fibroblast cells and skin. Both the in vitro and in vivo knockdown of short-chain fatty acid (SCFA) or free fatty acid receptor 2 (FFaR2) considerably blocked the probiotic effect of *S. epidermidis* on p-ERK-induced collagen type I induction. These results demonstrate that butyric acid (BA) in the metabolites of fermenting skin probiotic bacteria mediates FFaR2 to induce the synthesis of collagen through p-ERK activation. We hereby imply that metabolites from the probiotic *S. epidermidis* fermentation of CIN as a potential carbon source could restore impaired collagen in the dermal extracellular matrix (ECM), providing integrity and elasticity to skin.

## 1. Introduction

Dermal fibroblasts are the key cells involved in the production of the dermal extracellular matrix (ECM), playing a key role in wound healing; aiding in healing/scar formation; and maintaining the strength, flexibility, and resistance of skin. During skin aging, the major alteration can be seen in the collagen proteins in ECM in dermal fibroblasts [1,2]—that is, collagen type I, which accounts for approximately 80% of the total collagen in adult human dermis, while collagen type III, which predominates in the gastrointestinal (GI) tract and vascular connective tissues, represents 10% of the total collagen [3,4]. Mitogen-activated protein (MAP) kinase cascades have been implicated in a broad range of cellular responses to stimuli that result in the proliferation and differentiation of cells that are involved in ECM formation [5]. Previous studies have shown that the contraction of collagen matrices by fibroblasts under isometric tension resulted in the activation of extracellular signal-regulated kinase (ERK) signaling.

Metabolites from the human probiotic bacteria *Staphylococcus epidermidis* (*S. epidermidis*) fermentation of different carbon sources such as sugar or polymers have been found to protect skin from inflammation in response to stress [6,7]. The oral administration of probiotics could effectively heal wounds through the deposition of collagen on them [8]. Moreover, *S. epidermidis* has the ability to attach to materials coated with extracellular matrix proteins such as fibrinogen and collagen type I through GehD, an extracellular lipase [9]. Metabolic homeostasis is maintained by Free Fatty Acids (FFAs), which are widely known to modulate signaling receptor and gene expression by activating specific G-Protein Coupled Receptors (GPCRs) in pancreatic β cells, immune cells, white adipose tissue, the intestine, and several other tissues, as well as modulating immune regulation via inflammation regulation and the secretion of peptide hormones [10,11]. FFaR2 is known as G-protein coupled receptor 43 (GPR43), one of the members of GPCRs which are activated by short-chain fatty acids (SCFAs) [11,12]. SCFAs, the end product of fermentation by bacteria, have one to six carbons, which include acetate (C2), propionate (C3), butyrate (C4), isobutyrate (C4), 2-Methyl-butyrate (C5), and isovalerate (C5) [10,13,14,15]. Here, we reported that SCFAs such as butyric acid (BA), as one of the major metabolites produced by the *S. epidermidis* fermentation of polymer CIN in the skin, induces collagen type I production through phosphorylated extracellular signal-regulated kinase (p-ERK) activation via interaction with FFaR2. Thus, our results suggest that *S. epidermidis*-butyric acid-FFaR2-p-ERK could be a potential therapeutic target to protect against skin aging through collagen type I production.

## 2. Results

### 2.1. CIN as a Selective Fermentation Initiators (SFIs) for S. epidermidis

**Figure 1:**
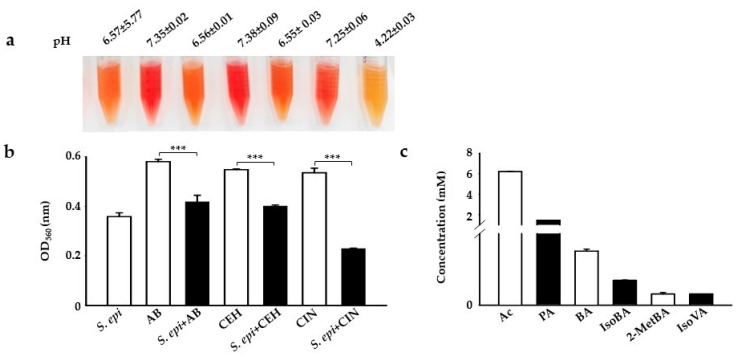
*S. epidermidis* mediates triggers CIN fermentation to produce SCFAs. (**a**) *S. epidermidis (S. epi)* (ATCC 12228) (10^7^ CFU/mL) was incubated in rich media containing phenol red with and without 2% AB, CEH, and CIN for 12 h. Rich media with phenol red plus 2% AB, CEH, or CIN were included as a control. The pH values of S. epi, AB, *S.epi*+AB, CEH, *S.epi*+CEH, CIN, and *S.epi*+CIN were 6.57 ± 5.77, 7.35 ± 0.02, 6.56 ± 0.01, 7.38 ± 0.09, 6.55 ± 0.03, 7.25 ± 0.06, and 4.22 ± 0.03, respectively. Bacterial fermentation was indicated by the color change of phenol red to yellow. (**b**) A graph showing the OD_560_ (nm) value in all the above groups. (**c**) The graph represents the amount of SCFAs generated from CIN fermentation by *S. epi* by GC-MS analysis. The *x*-axis represents the type of SCFAs used in the fermentation: Ac, acetic acid; BA, butyric acid; PA, propionic acid; IsoBA, isobutyric acid; 2MetBA, 2-methyl butyric acid; and IsoVA, isovaleric acid. The *y*-axis is their concentration in millimolars (mM). Data are expressed as means ± SD. (*** *p* < 0.001; *n* = 3).

Carbon sources or polymers such as Selective Fermentation Initiators (SFIs) can be easily screened for fermentation by bacteria using small-scale fermentation in specific fermentation media with phenol red as an indicator. It has been reported that bacteria can use a PEG polymer as a carbon source for fermentation, converting it to acetate, butyrate, or ethanol [16,17]. Here, we examined the selective fermentation of PEG analogs such as c_12–14_ alkyl benzoate (AB), cetyl ethylhexanoate (CEH), and CIN by incubating these SFIs with skin commensal *S. epidermidis* at 10^7^ colony-forming units per milliliter (10^7^ CFU/mL) in a rich medium with phenol red. *S. epidermidis*, AB, CEH, or CIN (2%) were included as a control. In agreement with previous results, the medium color changed from red to orange to yellow, which we confirmed by detecting a change in the power of hydrogen (pH) and optical density at (OD) 560 nanometers (nm)_._ A change in the color of phenol red in the media from red to yellow and the acidification of the media with a striking drop in pH (4.22 vs. 6.57 and 7.25 in controls) was observed in media containing bacteria with CIN as a carbon source for fermentation after 12 h of incubation [7,18,19,20]. Although there was detected to be a OD_560_ nm reduction in AB and CEH, a mild change in media color to orange with no significant change in pH (Figure 1a) was found in these carbon sources (6.56 vs. 6.57 and 7.35 in controls) (6.55 vs. 6.57 and 7.38 in controls) (Figure 1b). In a rich medium incubated with *S. epidermidis* alone, the color of phenol red changed from red to orange because of the bacterial replication during incubation. Next, we screened the supernatant following the CIN (2%) fermentation of *S. epidermidis* to quantify its SCFA producing capacity through gas chromatography-mass spectrometry (GC-MS) analysis [16]. Six SCFAs, including acetate, butyrate, propionate, iso-butyrate, 2-methyl-butyrate, and iso-valerate, were detectable in the media of the CIN fermentation of *S. epidermidis* (Figure 1c).

### 2.2. Mixture of CIN and S. epidermidis Induces the Expression of Collagen Type I and p-ERK Production in Mouse Skin

**Figure 2:**
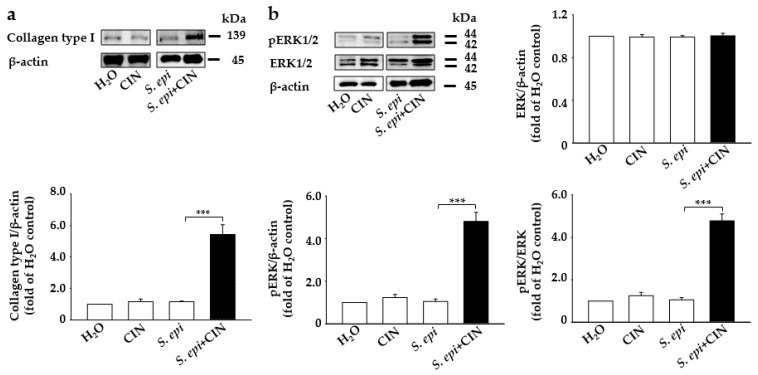
Application of a mixture of *S. epidermidis* and CIN induces collagen type I and p-ERK expression in mouse skin. Fermented media from *S. epidermidis* (S. epi) (ATCC 12228) (10^7^ CFU/mL) were topically applied to mouse skin in the presence and absence of CIN 2%. Mice treated with H_2_O or CIN alone were included as a control. Protein expression of (**a**) collagen type I and β-actin and (**b**) p-ERK 1/2, ERK 1/2, and β-actin from Western blot analysis in mouse skin treated with H_2_O, CIN, *S. epi*, and *S. epi*+CIN is shown. Ratios of intensities of collagen type I, p-ERK 1/2, and ERK 1/2 relative to β-actin and p-ERK1/2 relative to ERK 1/2 in all the above mice groups are illustrated. Data are expressed as means ± SD (*** *p* < 0.005; *n* = 3).

The degradation of the ECM has directly been linked to skin aging, with an increase in the activity of certain enzymes such as collagenases or matrix metalloproteinase. causing the deterioration of connective tissue proteins such as collagen in the primary human skin dermal fibroblasts, leading to a loss of strength and flexibility in the skin [21,22]. Studies have evidenced that probiotics accelerate the fibrosis process, causing the deposition of collagen [8]. A mixture of *S. epidermidis* (10^7^ CFU/mL) and CIN (2%) was topically applied to the dorsal skin of mouse for 1 week. Western blotting and densitometric analysis of protein bands from mouse skin showed a more than 3-fold increase in the expression of collagen protein in mouse skin treated with *S. epidermidis* plus CIN compared to controls treated with dihydrogen monoxide (H_2_O), *S. epidermidis*, or CIN (Figure 2a). Moreover, it has been documented that ERK regulation plays an important role in the regulation of collagen protein in human skin fibroblasts [3,23]. We detected an upregulated level of p-ERK1/2 in mouse skin treated with *S. epidermidis* plus CIN compared to control mice (Figure 2b). However, the protein level of total ERK1/2 remained unchanged in all groups. This indicates that metabolites from CIN fermentation with *S. epidermidis* are able to inhibit collagen type I degradation and induce p-ERK1/2.

### 2.3. Knocking Down FFaR2-Inhibited BA-Mediated Induction of Collagen Type I and p-ERK in Mouse Fibroblasts

**Figure 3:**
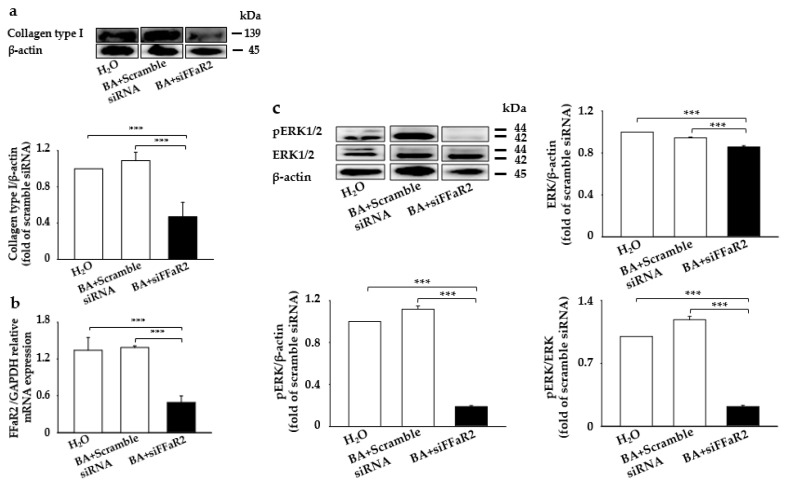
Blocking FFaR2 prevents BA-mediated collagen type I and p-ERK induction. 3T3 cells were transfected with FFaR2 (siFFaR2) or scrambled siRNA followed by treatment with and without BA for 24 h. 3T3 cells treated with H_2_O were included as a control. (**a**) Protein expression analyzed by the Western blot analysis of collagen type I and the ratio of intensities of collagen type I to β-actin. (**b**) The expression of the FFaR2 gene relative to the GAPDH gene by RT-PCR analysis. (**c**) Protein expression analyzed by the Western blot analysis of p-ERK1/2, ERK1/2, and β-actin and the ratio of intensities of pERK1/2 and ERK/2 relative to β-actin and the ratio of intensity of p-ERK1/2 to ERK1/2 in the above groups are displayed. Data are expressed as means ± SD (*** *p* < 0.001; *n* = 3).

BA, one of the SCFAs from the *S. epidermidis* fermentation of CIN, was found to have a potential role in the BA stimulation of MAP kinases and collagen biosynthesis in cultured human skin fibroblasts [23,24]. Moreover, it has been documented that BA mediates its chemoattractant anti-inflammation and immunoregulatory role by directly activating its cognate GPR43 (also known as FFaR2) [7]. To further evaluate the role of BA in collagen and p-ERK expression and interaction with its cognate FFaR2, we further knocked down FFaR2 with small interfering FFaR2 (siFFaR2) (5 µM) transfection into 3T3 mouse fibroblast cells, followed by treatment with BA. A significant upregulation in collagen type I and p-ERK was found upon the application of butyrate injected with scrambled small interfering ribonucleic acid (siRNA) compared to the control mouse treated with H_2_O alone (Figure 3). However, a notable decrease in collagen type I and p-ERK was detected in FFaR2-deficient 3T3 cells, which did not recover even after butyrate application (Figure 3a,c). We also confirmed the FFaR2 gene knockdown by measuring the relative expression of messenger ribonucleic acid (mRNA) by real-time polymerase chain reaction (RT-PCR) analysis and FFaR2 expression (Figure 3b and Figure S1). Additionally, the upregulated level of collagen expression upon the treatment of 3T3 cells with fermented media from CIN fermentation by *S. epidermidis* was downregulated upon knocking down FFaR2 (Figure S2). Studies on the effect of BA on collagen expression stated that collagen biosynthesis is regulated through ERK signaling in human dermal fibroblasts [23,25]. These results show that BA, the fermentation metabolite, induces collagen type I-pERK expression in mouse fibroblasts through BA–FFaR2 interaction.

### 2.4. Knocking Down FFaR2-Inhibited Fermentation Metabolite-Mediated Induction of Collagen Type I and p-ERK in Mouse Skin

**Figure 4:**
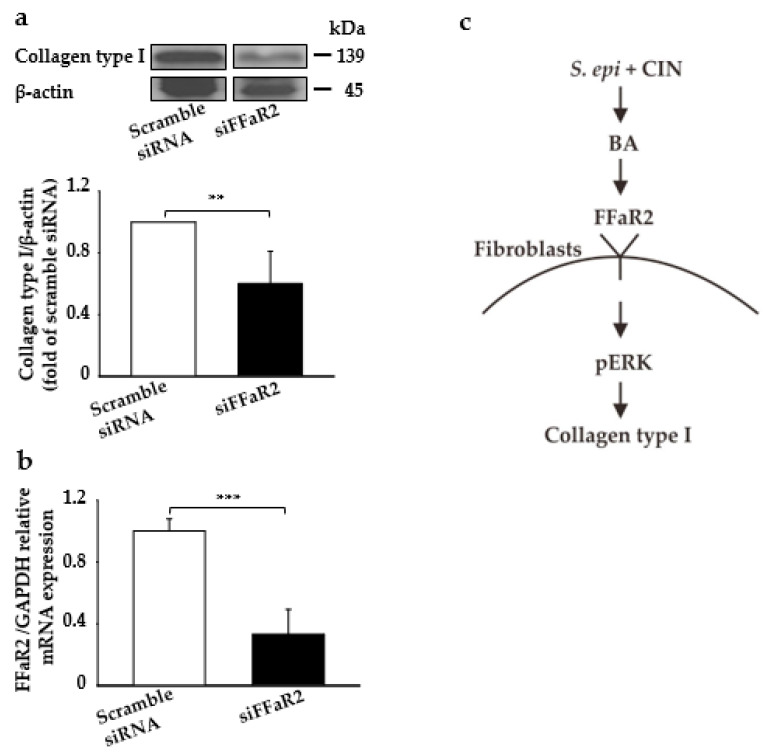
Blocking FFaR2 prevents fermentation-mediated collagen type I and p-ERK induction in a mouse skin model. Mouse skin was subcutaneously injected with FFaR2 (siFFaR2) or scrambled siRNA 20 min prior to the topical application of fermented media from *S. epidermidis* (ATCC 12228) (10^7^ CFU/mL) in the presence of CIN 2%. (**a**) Protein expression analyzed by the Western blot analysis of collagen type I and β-actin, and the ratio of intensities of collagen type I relative to β-actin. (**b**) The expression of the FFaR2 gene relative to the GAPDH gene as analyzed by RT-PCR analysis in mouse skin from all the above groups. (**c**) The schema of CIN 2% fermentation by S. epidermidis, which produces BA that further activates pERK1/2 via FFaR2 in fibroblast cells, ultimately stimulating the synthesis of collagen type I in a mouse skin model. Data are expressed as means ± SD (** *p* < 0.05; *** *p* < 0.005; *n* = 3).

Here, we detected the effect of the direct application of fermented media from the CIN fermentation of *S. epidermidis* on the induction of collagen type I production, ERK regulation, and their interaction with FFaR2. We blocked FFaR2 receptor via the subcutaneous injection of siFFAR2 (5 µM) into the dorsal skin of ICR mice 20 min prior to the topical application of fermented media from S. epidermidis with CIN. Mice injected with scrambled siRNA was included as a control. As shown in Figure 4a, the collagen type I content was significantly increased by the application of fermented media from the CIN fermentation of *S. epidermidis*, which was further attenuated if it compared to in FFaR2-deficient mice. The FFaR2 gene knockdown was further confirmed by measuring the mRNA relative expression through RT-PCR analysis and FFaR2 expression (Figure 4b and Figure S3). Thus, SCFAs such as BA, a metabolite from the CIN fermentation *of S. epidermidis*, interact with FFaR2 receptor, which further upregulates p-ERK and ultimately leads to collagen type I production in the downstream pathway (Figure 4c). Additionally, the collagen type I content was significantly decreased after FFaR2 was inhibited by via the gavage feeding of FFaR2 selective antagonist GLPG0974 (0.1–1 mg/kg) 20 min prior to the topical application of BA (Figure S4); this showed a similar effect in the knocking down of FFaR2 when targeting different regions (Figure S5).

## 3. Discussion

In the present study, we investigated whether BA, as a fermentation metabolite from the CIN fermentation of *S. epidermidis*, induces collagen biosynthesis and p-ERK through interaction with its cognate receptor FFaR2. Previous studies have shown that SCFAs induce the chemotaxis of neutrophils via the activation of FFaR2 [26]. FFaR2, which is highly abundant and responsive in neutrophils, can promote the production of collagen in fibroblast cells; however, an abundance comparison with FFaR2 and other counterparts (FFaR1, FFaR3, and FFaR4) has not been determined yet [26,27,28,29]. As the interest in anti-aging treatments has grown with the increase in life expectancy, much research has been dedicated to the development of products to prevent skin aging. Although aging is either intrinsic or extrinsic, intrinsic aging involves a reduction in the levels of ECM components, such as collagen type I, that the most predominant structural protein in fibroblasts of the skin epidermis [30]. In the skin of older humans, collagen type I is significantly decreased in the dermal fibroblasts, with thin collagen fibers compared to those in younger skin [31,32]. Taking high doses of growth hormone in the long term (for more than a few months) might lead to diabetes, high blood pressure, or heart disease [33]. Although target aging with metformin (TAME), which is a food and drug administration (FDA)-approved drug, is extensively used for targeting aging, its use is still restricted in healthy subjects without any health ailments such as diabetes or risk of developing cardiovascular disorders, which make it a limited drug of choice [34].

To date, oral and topical probiotics for the skin microbiome play an important role in treating inflammatory skin diseases, atopic dermatitis, acne, rosacea, wound healing, and skin cancer [7,35,36]. Numerous studies have found that there are higher number of *S. epidermidis* cells in the underarm (axilla) portion of the human skin than in the head and neck [37,38,39]. Meanwhile, a higher amount of collagen in the ECM is produced by fibroblast cells in the dermal layer of the skin [2,4,37,40]. Human skin aging contributes to a loss of collagen production and a loss of the amount of *S. epidermidis* due to intrinsic or extrinsic factors. Intrinsic factors include the natural aging of old human skin, with major collagen and *S. epidermidis* being significantly decreased in the dermal fibroblast compared to young skin, which shows thin collagen fibers and a high amount of *S. epidermidis* and extrinsic factors. This is associated with environment and lifestyle consequences, such as exposure to ultraviolet B (UVB) irradiation or reactive oxygen species (ROS), which trigger skin damage and decrease the number of *S. epidermidis* cells in the skin [31,32,41,42,43,44,45,46]. 131 alkyl PEG have been assessed by the cosmetic ingredient review (CIR) expert panel and their application in cosmetics at a specific concentration has been proven to be safe. Moreover, these polymers are formulated to be nonirritating and function in cosmetics as surfactants, skin-conditioning agents, and/or emulsifying agents [47,48].

PEG polymer, a biocompatible and biodegradable substance, is widely used in drug applications through transdermal penetration into the skin [49,50]. The stratum corneum constitutes an effective barrier and normally absorbs a few percent of topically applied doses [51]. A previous study reported that the pharmacokinetics-related methodologies and bioequivalence performance characteristics converge to promote rigorous bioequivalence requirements for topical dermatological drug products [52]. Additionally, the distribution of PEGylated proteins and PEG in tissues and cells relies on the size of the PEG proteins, the type of PEG used, and the potency of receptor-mediated PEG protein uptake. In the acidic environment of the lysosome, PEGylated proteins can be metabolized and metabolic products including PEG can be released from the cell [53], while the microbiota can mediated the metabolite of drugs by enzyme activity [54]. An extracellular enzyme was recognized to depolymerize PEG polymer [55]. BA, one of the SCFAs from the PEG polymer of metabolic products, is usually in a low range from 1 to 4 micromolar (μM) in peripheral circulation [56]. The pharmacokinetic study showed the butyrate metabolism in the mouse to have a half-life < 5 min [57]. Meanwhile, drug excretion primarily takes place in the liver and kidney. The kidney is the main organ involved in drug excretion. The renal clearance of drugs includes glomerular filtration, tubular secretion, and reabsorption [54]. Based on this, in the current study we used CIN, a polymer of PEG, as a selective carbon source for fermentation by the skin probiotic *S. epidermidis*; it can be used for emollients or medium polarity. These compounds are already enlisted in the international nomenclature of cosmetic ingredients (INCI) and are currently used as ingredients in various cosmetic products. The fermentation of CIN by *S. epidermidis* was detected by the change in color of phenol red from red to yellow (more acidic), which was further validated quantitatively by a significant decrease in the OD_560_ nm and pH within 12 h. This was influenced by one of the main enzymes involved in fermentation, acetolactate synthase (ALS), which converts pyruvate into 2-acetolactate and isobutyrate, and eventually into butyrate [58]. Our previous studies have shown a potential role of ALS activity in regulating the fermentation of carbon sources such as glycerol or sodium _L_-lactate by *S. epidermidis* [7,19].

Additionally, PEG and its polymers such as CIN as a carbon source have a physiochemical structure and outstanding flexible chain similar to that of alkyl PEG ethers, which facilitates its connection to enzymes [48], restoring the clumsiness and the balance of the enzyme, showing that CIN fermentation by *S. epidermidis* may be regulated by ALS activity. Similarly to earlier studies of the *S. epidermidis* fermentation of other carbon sources, CIN fermentation by *S. epidermidis* yielded a large amount of SCFAs such as acetate, propionate, butyrate, iso-butyrate, 2-methyl-butyrate, and isovalerate by GC-MS analysis [7,19]. Several lines of evidence suggest that metabolites from microbial fermentation—e.g., skin commensal *S. epidermidis* using PEG or PEG-like polymers—have been under investigation as potential therapeutics against different infectious and inflammatory diseases [16,59]. We found that the topical application of a mixture of *S. epidermidis* with CIN could noticeably induce collagen type I synthesis in mouse fibroblasts both in vitro and in vivo, suggesting that the anti-aging effect exerted through collagen type I induction observed here is likely to be mediated through CIN fermentation by *S. epidermidis.* A significant amount of BA was detected as a metabolite from fermentation CIN by *S. epidermidis* application, which effectively increased collagen biosynthesis in mouse fibroblasts, supporting the notion that BA as a potential microbial fermentation product regulates cellular growth and gene expression through the interaction between cell and ECM collagen proteins [60].

CIN exposure without *S. epidermidis* served as a negative control to show that a fermentation process using *S. epidermidis* is required to produce SCFA and induce collagen type I through the activation of FFaR2. A previous study showed that when the skin is wounded*, S. epidermidis* migrates from the skin surface to the dermal layer, which has a low content oxygen in the environment and undergoes the fermentation process using a carbon source to produce SCFA [61,62]. Interestingly, we can examine the sample from CIN applied topically in mouse skin, which was identified by GC-MS to identify SCFA products from the skin mouse sample for future study. Furthermore, the study evidenced that the activation of ERK and other MAP kinases is an essential event when human fibroblasts under isometric tension contract collagen matrices and a BA analog sodium butyrate (NaB), potentially regulating collagen biosynthesis and MAP-kinase expression [5]. A previous study found that mitogen-activated protein kinases/extracellular signal-regulated kinases (MEKs) are induced by an increase in p-ERK. Thus, MEK–pERK/ERK activation is involved in collagen-ECM production [5,63].

In the current study, the application of both CIN plus *S. epidermidis* or BA alone increased the p-ERK expression in mouse skin and fibroblast cells, although the total ERK expression remained unchanged. Moreover, the gavage feeding of mice with GLPG0974, an antagonist for FFAR2/GPR43 receptor, inhibited the effect of butyric acid, which led to a decrease in the collagen type I expression. Further, a study showed that SCFAs potentiates the mitogenic action of FFaR2 expressed in 3T3 cells, adipose tissue, intestines, islet cells of the pancreas, and immune tissues and plays an important role in maintenance of metabolism and homeostasis [10,11,13,64]. Additionally, BA could modulate immune/inflammatory reactions in the skin by via binding to its cognate receptor, FFaR2/GPR43 [7,65]. The notable increase in collagen or p-ERK in mouse skin and fibroblast cells was completely blunted by FFaR2 silencing using RNA interference [66]. Additionally, p-ERK effectively regulates collagen protein in human skin fibroblasts [23], indicating that probiotic *S. epidermidis* fermentation or BA as a metabolite from *S. epidermidis* upregulates p-ERK expression in the skin, ultimately leading to collagen type I induction through BA–FFaR2 interactions. These findings demonstrated the critical role of FFaR2 activation by SCFA from CIN fermented by *S. epidermidis*, a probiotic bacterium, potentially reducing the collagen type I-degradation in the skin, supporting the notion that the administration of probiotics could be a useful approach to counter aging.

## 4. Materials and Methods

### 4.1. Ethics Statement

The animal protocol used in this study was reviewed and approved by the Institutional Animal Care and Use Committee (IACUC) protocol of the National Central University (NCU), Taiwan (NCU-107-009), with respect to ethical issues and scientific care. Institute of Cancer Research (ICR) mice (8–9 week-old females; National Laboratory Animal Centre, Taipei, Taiwan) were sacrificed using 10% CO_2_ in a closed box [67].

### 4.2. Bacterial Culture

*S. epidermidis* bacteria, from ATCC 12,228 was cultured in tryptic soy broth (TSB) (Sigma, St. Louis, MO, USA) agar plates overnight at 37 °C. A single colony was inoculated in 3% TSB medium (Difco, Becton Dickinson UK Ltd., Oxford, UK) and further cultured at 37 °C. Overnight cultures were diluted 1:100 and cultured to an absorbance at 600 nm (optical density OD_600_) = 1.0. Bacteria were used for further experiments.

### 4.3. Fermentation of Bacteria

*S. epidermidis* (10^7^ CFU/mL) were incubated in 10 mL rich media (10 g/L yeast extract (Biokar Diagnostics, Beauvais, France), 3 g/L TSB, 2.5 g/L K_2_HPO_4_, and 1.5 g/L KH_2_PO_4_) in the presence of AB, CEH, and CIN (Taiwan NJC Corp., Chiayi, Taiwan), respectively, under aerobic conditions at 37 °C, with shaking at 200 rpm. The rich media with AB, CEH, and CIN without bacteria were included as a control. The 0.002% (*w/v*) phenol red (Sigma) in rich media acted as a fermentation indicator. A color change from red-orange to yellow indicated the occurrence of bacterial fermentation, which was detected at an absorbance of 560 nm.

### 4.4. GC-MS Analysis

*S. epidermidis* (10^7^ CFU/mL) was incubated in rich media with CIN 2% at 37 °C with shaking at 200 rpm. The fermentation media was centrifuged at 5000× *g* to remove *S. epidermidis*. The fermentation media was separately subjected to GC-MS analysis using an earlier published protocol [68].

### 4.5. Cell Culture

The adherent 3T3 (ATCC^®^ CRL-1658™) cells were cultured in Dulbecco’s Modified Eagle’s Medium (DMEM) high glucose (Gibco, Invitrogen, USA) media supplemented with 10% of fetal bovine serum (FBS) and 1% of penicillin/streptomycin/Amphotericin B Solution (P/S/A) (Biological Industries, Cromwell, CT, USA) at 37 °C, 5% CO_2_ and 95% humidity. The cells were grown until 90% confluence and then subcultured with Trypsin-Ethylenediamine tetra acetic acid (EDTA). Cells were seeded at a density of 8 × 10^5^/well in a 10 cm dish and the medium (10 mL) was replaced every 2 days.

### 4.6. Western-Blotting

Mouse dorsal skin was collected and around 100 mg of skin tissue was homogenized in 1 mL of hypotonic Tissue Protein Extraction Reagent (T-PER™) (ThermoFisher Scientific, Waltham, MA, USA). 3T3 cells were treated with and without BA for 24 h and the total protein was extracted using RIPA lysis Extraction Buffer (ThermoFisher Scientific) supplemented with a protease inhibitor cocktail (Sigma-Aldrich). Both tissue and cell lysates (40 µg) from different experiments were subjected to 10% sodium dodecyl sulfate (SDS) polyacrylamide gel electrophoresis with 4x Laemmli sample buffer (Bio-rad, Hercules, CA, USA). Electrophoresis-separated proteins were blotted onto polyvinylidene difluoride (PVDF) membrane (Millipore, Temecula, CA, USA) and were then incubated with primary antibody Collagen type I (1:2000, Cusabio Technology, Houston, TX, USA), ERK (1:1000, Cusabio), p-ERK (1:1000; Cell Signaling Technology, Danvers, MA, USA), and β-actin (1:5000, Cusabio), followed by secondary antibody—e.g., goat anti-rabbit immunoglobulin G (IgG) or donkey anti-goat IgG conjugated with horseradish peroxidase (ThermoFisher Scientific)—at a dilution of 1:2000. The immunoblots were visualized by adding chemiluminescence detection reagent (Bio-rad, Hercules, CA, USA) for 5 min and captured by the Omega Lum™ C Imaging System (Gel company, San Francisco, CA, USA). In some experiments, the first bound antibody-like collagen type I or p-ERK from the blots was removed by the stripping buffer (ThermoFisher Scientific), followed by washing with tris-buffered saline containing 0.1% tween 20 (TBST), and further incubation with the next antibody-like β-actin or ERK. Image data were analyzed using the Image J software (NIH, Bethesda, MD, USA), and normalized to β-actin.

### 4.7. siRNA-Mediated Gene Silencing of GPR43/FFaR2

The chemically modified siRNA target GPR43 receptor and the siRNA negative control were provided by GenePharma Co. (Shanghai, China). The oligonucleotide sequences are siFFaR2: sense strand, 5′-CCGGUGCAGUACAAGUUAUTT-3′; anti-sense strand, 5′-AUAACUUGUACUGCAC CGGTT-3′. siRNA targeting a different region/siFFaR2#1: sense strand, 5′-GGCACUGAGAACCAAAUAATT-3′; anti-sense strand, 5′-UUAUUUGGUUCUCAGUGCCTT-3′. SiControl: sense strand, 5′-UUCUCCGAACGUGUCACGUTT-3′; anti-sense strand, 5′-AACGUGACACGUUCGGAGAATT-3′. These chemically modified siRNAs were delivered intradermally by injection in mouse dorsal skin using a microneedle [7].

### 4.8. Drug Treatment

Selective FFaR2 antagonist GLPG0974 (0.1 or 1 mg/kg ig) was administered to ICR mice by gavage feeding [69]. GLPG0974 was dissolved in DMSO (0.01% in saline) and DMSO (0.01% in saline) was used as the vehicle control.

### 4.9. RT-PCR

The ICR mice’s skin was injected or treated with siRNA targeting scramble siRNA or siFFaR2 followed by topical application or treatment with fermented media of *S. epidermidis* with CIN 2% or BA within 24 h in mouse skin or 3T3 cells. The total RNA was isolated using the Purelink RNA mini kit (Invitrogen, USA) and homogenized to reduce the viscosity of difficult tissue samples (Invitrogen, USA). RNA (100 ng) was converted into complementary deoxyribonucleic acid (cDNA) using an iScript cDNA Synthesis Kit (Bio-Rad, Hercules, CA, USA). All sets were designed using the National Center for Biotechnology Information (NCBI) Primer-Blast (https://www.ncbi.nlm.nih.gov/tools/primerblast/). The reaction was performed on StepOnePlus RT- PCR System (ThermoFisher) using Power SYBRGreen PCR Master Mix (Thermo Fisher Scientific). The reaction conditions for 40 cycles are as follows: 95 °C for 10 min followed by 95 °C for 15s, 55 °C for 60 s, and 72 °C for 30 s. The expression of glyceraldehyde 3-phosphate dehydrogenase (GAPDH) gene was used for normalization. The levels of relative expression levels were calculated using the cycle threshold (2-∆∆Ct) method [70,71]. The primers used for FFAR2 and GAPDH were 5′-ACCCAAGAGCAGCTGGATGT-3′ (forward); 5′-AGCGCCTAACAGAAGATGGT-3′ (reverse) and 5′-TGTGTCCGTCGTGGATCTGA-3′ (forward); 5′-GATGCCTGCTTCACCACCTT-3′ (reverse), respectively.

### 4.10. Statistical Analysis

Data were represented as mean ± standard deviation (SD) from three independent experiments. An unpaired t-test or by one-way ANOVA using the SigmaStat (Jandel Scientific, Palo Alto, CA) software was performed to test statistical significance. The *p* values of <0.5 (*), <0.01 (**), and <0.001 (***) were considered statistically significant. The mean ± SD for at least three independent experiments was calculated.

## Data Availability

The data that support the findings of this study are available from the corresponding author upon reasonable request.

## Supplementary Materials

Supplementary Materials can be found at https://www.mdpi.com/1422-0067/22/3/1414/s1.

## Abbreviations

AB	C_12-14_ alkyl benzoate
Ac	Acetic acid
ALS	Acetolactate synthease
ATCC	American type culture collection
BA	Butyric acid
BSA	Bovine serum albumin
cDNA	Complementary deoxyribonucleic acid
CEH	Cetyl ethylhexanoate
CFU	Colony forming unit
CIN	Cetearyl isononanoate
CIR	Cosmetic ingredient review
CO_2_	Carbon dioxide
H_2_O	Dihydrogen monoxide
DMEM	Dulbecco’s modified eagle’s medium
DMSO	Dimethyl sulfoxide
ECM	Extracellular matrix
EDTA	Ethylenediamine tetraacetic acid
ERK	Extracellular signal regulated kinase
FBS	Fetal bovine serum
FDA	Food and drug administration
FFaR2	Free fatty acid receptor 2
FFAs	Free fatty acids
GAPDH	Glyceraldehyde 3-phosphate dehydrogenase
GC-MS	Gas chromatography-mass spectrometry
GPCRs	G-protein coupled receptors
GPR43	G-protein coupled receptor 43
H	Hour
IACUC	Institutional animal care and use committee
IgG	Immunoglobulin G
INCI	International nomenclature of cosmetic ingredients
IsoBA	Isobutyric acid
IsoVA	Isovaleric acid
kDa	Kilodalton
MAP	Mitogen-activated protein
MEK	Mitogen-extracellular signal regulated kinase
MMP	Matrix metalloproteinase
2MetBA	2-Methyl butyric acid
Min	Minute
mRNA	Messenger ribonucleic acid
μM	Micromolar
NaB	Sodium butyrate
NCBI	National center for biotechnology information
NCU	National Central University
NM	Nanometer
OD	Optical density
PA	Propionic acid
PBS	Phosphate buffer saline
PEG	Polyethylene glycol
p-ERK	Phosphorylated extracellular signal regulated kinase
pH	Power of hydrogen
PVDF	Polyvinylidene difluoride
ROS	Reactive oxygen species
RNA	Ribonucleic acid
RT-PCR	Real time polymerase chain reaction
*S. epidermidis*	*Staphylococcus epidermidis*
SCFAs	Short chain fatty acids
SD	Standard deviation
SDS	Sodium dodecyl sulfate
SFIs	Selective fermentation initiators
siFFaR2	Small interfering free fatty acid receptor 2
siRNA	Small interfering ribonucleic acid
TAME	Target aging with metformin
TBST	Tris-buffered saline tween 20
T-PER	Tissue protein extraction reagent
TSB	Tryptic soy broth
UVB	Ultraviolet B
