# Supplementary material for: Probiotic Activity of Staphylococcus epidermidis Induces Collagen Type I Production through FFaR2/p-ERK Signaling
Source: Int J Mol Sci. 2021 Jan 31;22(3):1414. doi: 10.3390/ijms22031414 (PMC7866835; doi:10.3390/ijms22031414)
Supplement: Supplementary file 1 [file ijms-22-01414-s001.pdf]

International Journal of Molecular Sciences

Article

Probiotic activity of *Staphylococcus epidermidis* induces collagen type I production through FFaR2/p-ERK signaling

Indira Putri Negari[1], Sunita Keshari[2] and Huang Chun-Ming[1,*]

1 Department of Biomedical Sciences and Engineering, National Central University, Taoyuan 32001, Taiwan; indiraputri24@g.ncu.edu.tw

2 Department of Life Sciences, National Central University, Taoyuan 32001, Taiwan; sunitakeshari827@gmail.com

* Correspondence: chunming@ncu.edu.tw; Tel.: +886-3-422-7151 (ext. 36101); Fax: +886-3-425-3427

1. Supplementary Information

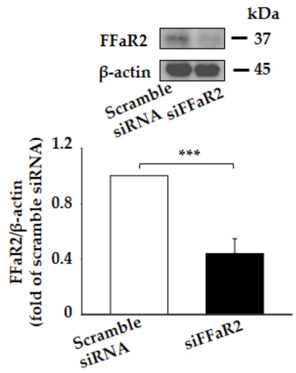

Figure S1. Blocking FFaR2 prevents the butyric acid mediated collagen and phosphorylated ERK induction. NIH-3T3 cells were incubated in the presence of butyric acid (BA) followed by treatment with FFaR2 (siFFaR2) or scrambled siRNA for 24 h. Western blot analyses of FFaR2 and the amount of FFaR2 was then expressed after normalization to β-actin, Data are expressed as means ± SD (****p*<0.001; n=3).

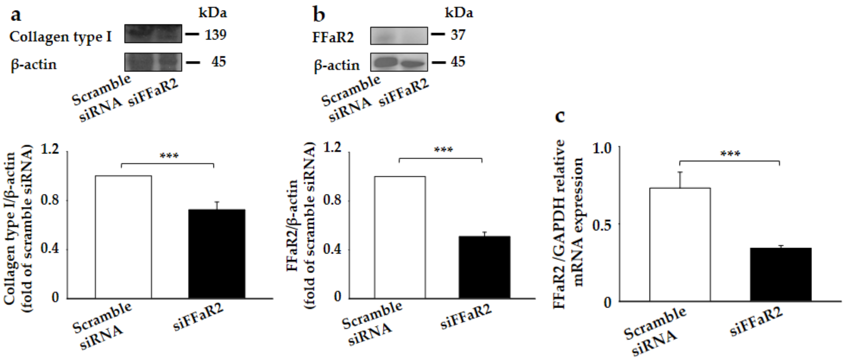

Figure S2. Blocking FFAR2 prevents the fermentation mediated collagen induction in NIH 3T3 cells. NIH-3T3 cells were incubated with fermented media from Staphylococcus epidermidis (ATCC 12228) (10^7 CFU/mL) in presence of Cetearyl isononanoate (CIN) 2% followed by treatment with FFaR2 (siFFaR2) or scrambled siRNA for 24 h. Western blot analyses of collagen type I (a), FFaR2 and β-actin (b) and the amount of collagen, FFaR2 were then expressed after normalization to β-actin. (c) The expression of the FFaR2 gene relative to the GAPDH gene by RTPCR analysis in mouse skin from all above groups. Data are expressed as means ± SD (***p<0.005; n=3).

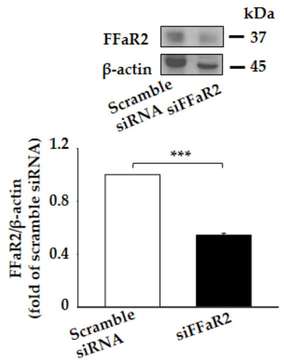

Figure S3. Blocking FFaR2 prevents the *fermentation* mediated collagen induction in mouse skin model. Mice skin were subcutaneously injected with FFaR2 (siFFaR2) or scrambled siRNA 20 min prior to topical application with fermented media from *Staphylococcus epidermidis* (ATCC 12228) (10^7 CFU/mL) in presence of Cetearyl isononanoate (CIN) 2%. (a) Western blot analyses of FFaR2 and β-actin, FFaR2 was then expressed after normalization to β-actin. Data are expressed as means ± SD (***p<0.001; n=3).

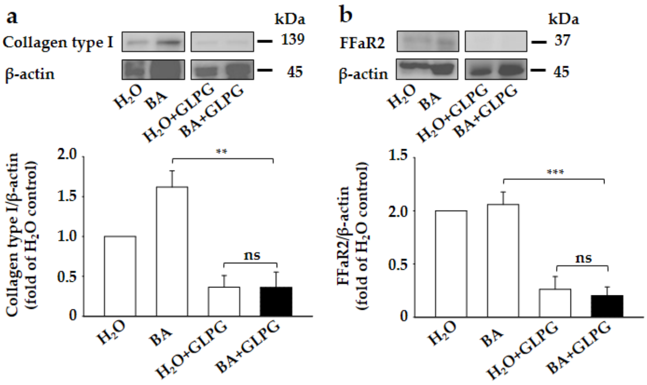

Figure S4. Induction of collagen content by application of BA. Mice skin were topically applied BA in the presence and absence of GLPG, an antagonist FFaR2. Mouse applied H_2O was included as control. Western blot analyses of collagen type I (a), FFaR2 and β-actin (b) and the amount of collagen, FFaR2 were then expressed after normalization to β-actin. Data are expressed as means ± SD (*p<0.5; ***p<0.001; n=3).

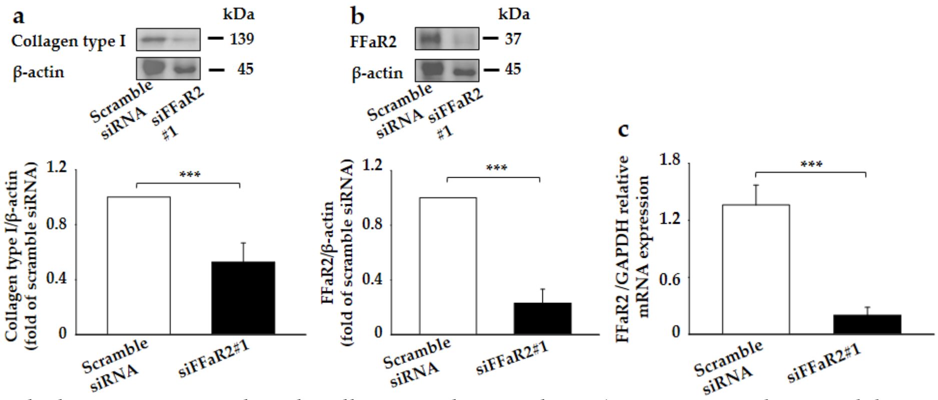

Figure S5. Blocking FFaR2 mediated collagen induction by BA in mouse skin model. Mice skin were subcutaneously injected with FFaR2-specific siRNA targeting a different region (siFFaR2#1) or scrambled siRNA 20 min prior to topical application with BA. Western blot analyses of collagen type I (a), FFaR2, and β-actin (b), and the amount of collagen type I and FFaR2 were then expressed after normalization to β-actin. (c) The expression of the FFaR2 gene relative to the GAPDH gene by RTPCR analysis in mouse skin from all above groups. (***$p<0.005$; n=3).
